# Pre-Service Teachers’ Perceptions of AI Technological Support and School-Based Intelligent Environment Support: Model-Based Indirect Associations with Innovative Competence via AI Self-Efficacy and Self-Regulated Learning

**DOI:** 10.3390/bs16081378

**Published:** 2026-08-11

**Authors:** Xu Liu, Meiqi Zhang, Jiaoyang Du

**Affiliations:** Center for Teacher Education Research, Faculty of Education, Beijing Normal University, Beijing 100875, China; liuxu.phd@mail.bnu.edu.cn (X.L.); 202221010132@mail.bnu.edu.cn (M.Z.)

**Keywords:** innovative competence, AI technological support, school-based intelligent environment support, AI self-efficacy, self-regulated learning, pre-service teacher

## Abstract

As artificial intelligence (AI) becomes deeply integrated into teacher education, pre-service teachers’ innovative competence has become a cornerstone for fostering human–AI collaborative innovation and enabling intelligent educational transformation. This study used empirical data from 3003 pre-service teachers across 12 provinces in China. It employed difference tests, quantile regression, and structural equation modeling to examine the associations of AI technological support and school-based intelligent environment support with pre-service teachers’ innovative competence. Results showed that pre-service teachers’ innovative competence was at a medium-to-high level, with significant heterogeneity across individual, family, and school background variables. Both supports were positively associated with innovative competence. However, the patterns of association differed. The association between AI technological support and innovative competence generally increased across the middle-to-upper quantiles, whereas the association between school-based intelligent environment support and innovative competence was stronger at the lower-to-middle quantiles and subsequently declined. AI self-efficacy and self-regulated learning both showed significant model-based indirect associations linking the two supports with innovative competence. Specifically, under AI technological support, AI self-efficacy had a larger mediating association than self-regulated learning; under intelligent environment support, the opposite pattern emerged. This study systematically examined the associations and mediating paths of AI technological support and intelligent environment support with pre-service teachers’ innovative competence. These findings identify correlational patterns consistent with the proposed framework and provide empirical evidence for optimizing AI-empowered teacher innovation cultivation.

## 1. Introduction

In the evolving integration of artificial intelligence (AI) and pre-service teacher education, the dual learner-educator identity of pre-service teachers creates a powerful transmission mechanism for innovation (Avsec & Savec, 2021; C. C. Liu et al., 2025); their own innovative competence directly shapes their ability and willingness to cultivate innovation in future students, thereby driving systemic transformation in talent development and ultimately strengthening the educational foundations of national competitiveness. International teacher education policy increasingly emphasises the integration of technology and teacher innovative competence. In the United Kingdom, for example, the recently published Initial Teacher Training and Early Career Framework explicitly states that, while technological tools are constantly evolving, teachers must remain conversant throughout their careers with technological developments that evidence suggests can improve pupil outcomes (Department for Education, 2024, p. 5). The framework further emphasises that providers of initial teacher training should support pre-service teachers in the judicious use of technology, informed by evidence, both in helping pupils to progress and in enabling and enhancing their own working practices (Department for Education, 2024, p. 5). Similarly, the Organisation for Economic Co-operation and Development (OECD), in its report Teaching Compass: Reimagining Teachers as Agents of Curriculum Change, notes that the relationship between teachers and AI tutors/copilots creates opportunities for coordinated instruction (OECD, 2025, p. 19). Within this emerging human-AI collaborative teaching landscape, the report contends that teachers require innovative capacity—namely, the competence to think innovatively, generate original ideas, make intuitive leaps, and draw on emotion, context, and experience to drive teaching practice (OECD, 2025, p. 21).

To systematically improve the quality of teacher education and establish new models for teacher preparation, China’s Ministry of Education and the National Development and Reform Commission jointly issued the Notice on Implementing the Teacher Education Capacity Enhancement Project in 2025. The Outline of the Plan for Building China into a Leading Country in Education (2024–2035) further articulates the strategic imperative to deepen the role of artificial intelligence in advancing teacher workforce development and professional competence. However, increasingly complex educational contexts, the iterative evolution of intelligent technologies, and the rapid growth of innovation culture pose significant challenges for pre-service teachers in navigating future educational roles while simultaneously raising the bar for their innovative competence. China’s approach to AI-integrated teacher education for pre-service teachers is currently at a critical juncture of initial exploration and technological integration and upgrading. AI-enabled teacher innovation development continues to face multiple difficulties, including the limited professional competence of AI-literate teachers (Ding et al., 2023), inadequate practical competence in human-AI collaborative education (F. Yang et al., 2024), and cognitive and experiential limitations regarding AI (Y. J. Zhang, 2025). Furthermore, the AI-related support systems for teacher preparation require urgent improvement (W. W. Li, 2024; Zhao et al., 2022b).

In light of this, this study examines the associations between AI support and pre-service teachers’ innovative competence, as well as the model-based indirect associations involving AI self-efficacy and self-regulated learning, thereby providing theoretical support and practical guidance for cultivating an innovative teaching workforce in the era of artificial intelligence.

## 2. Literature Review

### 2.1. The Relationship Between AI Support and Pre-Service Teachers’ Innovative Competence

As teachers are the implementers and leaders of innovative education in schools, their innovative competence constitutes a fundamental prerequisite for implementing innovative education and a critical guarantee for cultivating innovative talents (S. Q. Huang, 2000). In the context of basic education, teachers’ innovative work behaviour is regarded as a key factor in driving educational reform and development. It refers to the innovative thinking and behavioural patterns that teachers demonstrate in the educational process, encompassing both the generation of new pedagogical ideas and concepts, as well as the capacity to translate innovative ideas into actual teaching practice. Research has indicated that teachers’ instructional innovative competence comprises four core elements: learning competence, pedagogical competence, social competence, and competence in modern educational technology (Cai et al., 2012), which collectively constitute the core structure of teacher innovative competence. In the TALIS conceptual framework released in 2018, teacher innovation is defined to encompass teachers’ innovative competence and behaviours across multiple dimensions, including pedagogical methods, technology application, personal attitudes, and school culture. Specifically, the framework conceptualizes teacher innovation from the perspectives of innovative teaching practices and school innovative climate, utilizing indicators including innovation readiness, technology integration, personal innovation, innovation attitudes, and innovation climate (Ainley & Carstens, 2018). However, teachers’ innovative competence in the era of artificial intelligence requires deep integration with technology. Therefore, this study adopts a technology-empowered conceptualisation of teacher innovation (B. M. Li et al., 2024; G. X. Wu, 2021). In this context, pre-service teachers’ innovative competence refers to a dynamic system of competences comprising the interaction of innovation willingness, innovation readiness, and innovation practice, aimed at effectively promoting the development of learners’ innovative thinking. Within this framework, innovation willingness denotes pre-service teachers’ proactive acceptance of new technologies and their belief in their own capacity to organically integrate these technologies into innovative teaching processes; innovation readiness refers to the value guidance, capacity building, and content preparation that pre-service teachers need to conduct technology-supported innovative teaching; and innovation practice refers to pre-service teachers’ actual implementation of technology-empowered innovations in teaching methods, pedagogical processes, and professional development.

This construct should be distinguished from several adjacent but non-equivalent competences. AI literacy centres on the informed, critical, and responsible understanding, evaluation, and use of AI (Long & Magerko, 2020; Gavriilidou, 2025). Digital competence refers to the confident, critical, and creative use of information and communication technologies to achieve goals in work, employability, learning, leisure, inclusion, and social participation (Ferrari, 2013). Technological Pedagogical Content Knowledge (TPACK) emphasises the integrated knowledge required to connect technology, pedagogy, and subject matter in teaching practice (Mishra & Koehler, 2006). By contrast, pre-service teachers’ innovative competence in the present study refers to their professional capacity to generate and implement novel and effective teaching ideas, instructional designs, teaching strategies, and research approaches. AI provides an important contextual condition and enabling resource for this competence; however, innovative competence is neither a subdimension of nor a direct outcome of AI literacy, digital competence, or TPACK. Rather, it is a conceptually related but independent construct that concerns how pre-service teachers translate AI-enabled possibilities into pedagogical innovation. Although all four constructs involve the appropriate use of technology, AI literacy, digital competence, and TPACK primarily emphasise technology-related knowledge and skills. In contrast, innovative competence concerns the integrated process of innovation willingness, innovation readiness, and innovation practice, with a focus on innovative outcomes. Accordingly, the present study adopts innovative competence as the focal outcome variable. AI literacy, digital competence, and TPACK are discussed only to clarify their conceptual boundaries and are not included as outcome variables in the empirical model.

In the present study, AI support is used as a broad term to encompass two complementary predictors: AI technological support and school-based intelligent environment support. This twofold distinction is grounded in the operational content of our measures and informed by prior research that differentiates teachers’ perceptions of technology-related factors from their perceptions of school-level enabling conditions (Gil-Flores et al., 2017; Shen et al., 2024). Importantly, both constructs were assessed through pre-service teachers’ self-reports and therefore represent participants’ perceptions and evaluations rather than objective, externally verified measures of technological provision or school-level conditions. AI technological support refers to participants’ perceived instrumental and functional affordances of AI technologies for learning, instructional design, teaching practice, and professional development, including their evaluations of effective AI use, AI-supported innovation, efficiency enhancement, task simplification, and interdisciplinary learning. School-based intelligent environment support refers to participants’ perceived organisational, infrastructural, and sociocultural conditions within schools that facilitate access to and use of AI-related resources, including adequate AI resources, functional intelligent education platforms, learning resources, internal and external collaboration networks, and encouragement to explore AI-use strategies collaboratively. Accordingly, AI technological support captures perceived functional affordances of AI technologies, whereas school-based intelligent environment support captures perceived enabling school conditions through which these affordances are accessed and enacted. The following paragraphs synthesise prior research on the relationships of AI technological support and school-based intelligent environment support with pre-service teachers’ innovative competence.

Regarding the relationship between AI technological support and pre-service teachers’ innovative competence, the application and evolution of artificial intelligence technologies in education have opened new avenues for cultivating such competence among pre-service teachers. AI has been discussed as being associated with instructional design, teaching environments, pedagogical innovative thinking, and pre-service teachers’ innovative work performance and overall competence. This ultimately cultivates a greater number of high-quality, professional, and innovative teachers suited to the new era.

First, AI technology can facilitate pre-service teachers’ instructional innovation. Intelligent tutoring systems, by tailoring learning materials and exercises to students’ progress and comprehension, assist pre-service teachers in more effectively grasping student learning situations and designing targeted instruction (Jia et al., 2023). Furthermore, pre-service teachers are better able to actively construct knowledge and achieve understanding and internalisation of instructional design theories when prompted by generative AI (L. Wu et al., 2024). Second, technological development drives the transformation and upgrading of classroom teaching environments (D. Wu et al., 2022). AI technology can simulate authentic teaching scenarios, allowing pre-service teachers to engage in teaching practice within virtual environments (Sun et al., 2024). For instance, by analysing micro-skills such as pre-service teachers’ instructional language, posture, expressions, and board design, AI can provide immediate feedback and suggestions for improvement, thereby helping them optimise their teaching performance. Leveraging virtual reality and augmented reality technologies, along with the development of supporting resources, can effectively enrich pre-service teachers’ teaching experiences and enhance their ability to respond flexibly to problems in instructional contexts (Song et al., 2023). This “virtual microteaching” model enables pre-service teachers to transcend temporal and spatial limitations, practice every aspect of classroom teaching, explore diverse teaching concepts, methods, and strategies, engage in repeated practice and trial and error, and subsequently reflect upon and improve their teaching (M. Lin et al., 2024).

Furthermore, AI technology demonstrates significant potential in cultivating pre-service teachers’ innovative pedagogical thinking. The deep integration of technology and education inherently entails the renewal and reshaping of ideologies, requiring pre-service teachers to continuously transcend existing conceptual boundaries and persistently advance the development and progress of educational ideas (Y. L. Qi & Zhou, 2024). Drawing on the driving factors of interdisciplinary innovative thinking, one study analysed the core functions of generative AI in empowering the cultivation of such thinking from the perspectives of efficient information retrieval and integration, multi-turn dialogue and role simulation, evaluation and feedback, and generation and creation, subsequently constructing a corresponding instructional model (Y. Dong & Chen, 2024).

Finally, AI has been discussed as potentially supporting the development of innovative competence. Specifically, with respect to innovation readiness, AI can facilitate the generation of novel ideas and innovative solutions by processing vast amounts of information and integrating cutting-edge developments and creative concepts (Bahoo et al., 2023). Regarding the emergence of innovation willingness, AI can provide individuals with a range of innovative solutions and space for exploration when addressing problems (Kakatkar et al., 2020). In terms of the development of innovation willingness, AI can assist individuals in accomplishing more challenging, complex, and creative tasks. When AI enhances an individual’s creative self-efficacy and innovative role identity within the creative process, it holds the potential to fundamentally transform their innovative personality (H. L. Wang et al., 2024). In summary, AI technology plays a significant role in pre-service teachers’ learning and professional development. It not only technologically empowers their instructional design capacity and drives the transformation and upgrading of teaching environments but also plays a crucial role in cultivating their innovative thinking.

School-based intelligent environment support is also significantly associated with pre-service teachers’ innovative competence. Drawing on the framework of the OECD Innovative Learning Environments project, the learning environment has been conceptualized as a complex system encompassing multiple key factors, including learners, teachers, learning content, learning organization, and resources (Roberts & Owen, 2012). For pre-service teachers, school-based intelligent environment support comprises the organisational, infrastructural, and sociocultural conditions within both their training institutions and practicum settings, involving elements such as teacher educators, mentor teachers, learning content, learning organization, and resources. At the organizational leadership level, research suggests that transformational leadership, school learning climate, and leader-member relationships influence teacher innovation (Vermeulen et al., 2022). Specifically, transformational leadership indirectly encourages teachers’ innovative behaviour by fostering a supportive learning climate and high-quality leader–member relations. In addition, a positive school climate, effective teamwork, and a collaborative culture that encourages cooperation, continuous learning, and open communication all foster teacher innovation (Noviyanti et al., 2021; Giles & Hargreaves, 2006). Together, these factors support innovation by enabling resource sharing, knowledge complementarity, and collective problem-solving. Regarding resources, studies have categorized them into four types: learning facilities, environmental characteristics, equipment and technology, and general resources. Notably, teachers require adequate resources and facilities to demonstrate innovative behavior (Thurlings et al., 2015). Therefore, providing sufficient and accessible technological training and professional development opportunities for pre-service teachers can effectively enhance their innovative competence. Based on the above, the research hypotheses are presented below in the literature review section.

### 2.2. The Mediating Role of AI Self-Efficacy

Regarding the relationship between AI technological support and pre-service teachers’ AI self-efficacy, self-efficacy generally refers to an individual’s assessment and judgment of their capability to successfully organize and complete tasks (Bandura, 1986). Specifically, pre-service teachers’ AI self-efficacy denotes their self-assessment of the ability to successfully use AI to organize and accomplish educational and teaching tasks. In the AI era, teachers’ technology anxiety (Johnson & Verdicchio, 2017), which includes apprehension about applying intelligent technologies, resistance to educational AI, and discomfort in “AI + education” environments, hinders their motivation to use AI. This anxiety is frequently associated with lower AI self-efficacy. Low AI self-efficacy not only impairs the effectiveness of AI use during pre-service teachers’ learning phase but also negatively impacts the sustainable development of AI-enabled teaching practices in their future careers. The primary reasons for teachers’ low perceived self-efficacy in using AI technology include unfamiliarity with and imprecise attitudes towards its application. Adequate support for AI technology use can enhance technology perception, mitigate technology anxiety (Zhao et al., 2022a), and thereby boost teachers’ AI self-efficacy. Experimental research has shown that teachers using generative AI technology scored significantly higher on measures of teaching self-efficacy and higher-order thinking skills compared to the control group (Lu et al., 2024), indicating that the use of AI technology can effectively increase pre-service teachers’ confidence and higher-order thinking abilities. For pre-service teachers, the perceived ease of use, usefulness, and concerns regarding data privacy and security of AI technology tools are significant factors influencing their willingness and confidence to use them (Davis, 1989). Intuitive user interfaces and simplified operational procedures can effectively reduce usage barriers and increase successful experiences, thereby enhancing pre-service teachers’ willingness and confidence in using AI tools (Legris et al., 2003). Furthermore, the actual effectiveness of AI tools in helping pre-service teachers improve instructional quality and facilitate student learning is another critical factor influencing their sustained use of AI technology (Koedinger & Corbett, 2006). If AI tools effectively meet teachers’ developmental needs, they increase pre-service teachers’ acceptance of AI, consequently elevating their AI self-efficacy. Additionally, robust data encryption for storage, transmission, and processing, along with transparent privacy policies (Cavoukian, 2008), is crucial for building pre-service teachers’ trust in AI technology.

Regarding the relationship between school-based intelligent environment support and pre-service teachers’ AI self-efficacy, such support constitutes a key external factor influencing their opportunities for and perceived efficacy in using AI. If teacher education institutions and practicum schools provide necessary AI resource support that is physically accessible and economically affordable, pre-service teachers are more likely to utilize AI technology for learning and teaching practice. Furthermore, school-based intelligent environment support encompasses policy backing, teacher education curriculum integration, and a supportive school culture. Leadership styles, interpersonal collaboration, and the overall school culture can convey positive messages about AI’s role in teacher development and encourage pre-service teachers, through exemplary modeling, to actively engage with AI technologies. Teacher education curricula also emphasize cultivating a new profile of teachers with attributes such as creativity, innovation, and collaboration, continuously enhancing their ability to creatively solve complex problems and engage in interdisciplinary innovative learning (X. H. Yu, 2020). This focus boosts pre-service teachers’ sense of accomplishment and competence, thereby promoting their AI self-efficacy. The satisfaction of basic psychological needs, such as achievement, competence, and self-efficacy, can motivate individuals to produce more positive behavioral outcomes (L. H. Liu & Zhang, 2010), which in turn influences the development of their innovative competence.

A high sense of AI self-efficacy among pre-service teachers is not only positively associated with their willingness and behavior to use AI but may also be positively associated with their innovative competence. Creativity is defined as an intellectual quality that fully mobilizes all resources to produce something novel, unique, and of social or personal value (C. D. Lin et al., 2009, p. 2). The notion of “fully mobilizing” indicates that innovative competence is a highly internalized literacy, requiring the support of psychological qualities such as strong self-efficacy, resilience, and an autonomous orientation. Research on the mechanism by which generative AI fosters creative potential also suggests that a curious and persistent personality is a distinctive manifestation of AI-derived creative potential, while interpersonal collaboration between teachers and students, and among students themselves, serves as a key guarantee for optimizing human–AI co-creative outcomes (C. L. Wang et al., 2024). An individual teacher’s openness to innovation refers to their innovation-related self-efficacy (Nemeržitski et al., 2013). Teachers with high self-efficacy are more open to innovative experiences and more willing to implement innovations than those with low self-efficacy (Tschannen-Moran & Hoy, 2001). Research on teachers’ innovative behavior has found that after incorporating the variable of innovative self-efficacy, it exerts a significant positive influence on cognitive activation and the enhancement of proactive innovative behavior (Y. B. Dong & Liu, 2024). This suggests that pre-service teachers’ AI self-efficacy may be positively associated with their innovative competence.

In summary, teachers’ innovative competence is positively associated with a combination of internal and external factors, including the use of information technology (van Merriënboer & Brand-Gruwel, 2005), school teaching culture (S. L. Wang & Zhou, 2013), innovation climate (Xia et al., 2019), innovation support (Cai & Gong, 2019), and principal leadership styles (S. L. Huang et al., 2005). As future teachers, pre-service teachers’ innovative competence is similarly shaped by the interaction of internal individual factors and external environmental factors. Individual cognitive appraisal serves as a direct antecedent that motivates behavior and fosters innovative competence. Organizational environmental factors, such as school climate and leadership, promote teacher innovation by providing support and resources, establishing platforms for collaboration and communication, and cultivating a positive organizational culture. Therefore, both AI technological support and school-based intelligent environment support are likely to be associated with innovative competence. However, their associations with pre-service teachers’ innovative competence may be fully realized only through the mediation of internal microsystem factors. Individuals with high self-efficacy often exhibit more positive learning attitudes and greater persistence, which in turn positively predict the development of their learning qualities (Alhadabi et al., 2019). Furthermore, self-efficacy plays a mediating role in the relationship between environmental support and self-regulated learning with intelligent technology (Gou et al., 2024). As a component of pre-service teachers’ personal value expectations, self-efficacy influences their technology-enabled teaching and innovative behavior as an intrinsic factor. Both the intelligent environment in school and personal disposition toward AI support affect competency achievement by influencing learners’ self-efficacy (Pekrun et al., 2002). Based on the above synthesis, the research hypotheses are proposed subsequently.

### 2.3. The Mediating Role of Self-Regulated Learning

In terms of the relationship between AI technological support and pre-service teachers’ self-regulated learning, self-regulated learning involves planning task content and methodological approaches, recording the learning process, adjusting strategies and goals, and reflecting on and synthesising experiences based on forethought, performance, and self-reflection (Zimmerman, 2000). As learners, pre-service teachers’ self-regulated learning refers to the process of continuously adjusting, organising, monitoring, and controlling their own cognition, motivation, and behaviour in response to changes in the school and higher education environment in order to achieve specific objectives. In the context of artificial intelligence, AI technological support can effectively facilitate learners’ self-regulated learning willingness and behaviour. Experimental research on undergraduate students’ use of AI technologies has shown that AI-assisted learning tools, such as ChatGPT (gpt-3.5-turbo-16k, OpenAI, San Francisco, CA, USA), can effectively reduce students’ technological dependence, enhance their level of self-regulated learning, and positively influence their higher-order thinking and knowledge construction (H. Y. Lee et al., 2024). Studies on English learning among university students have also shown that AI-supported gamified learning can promote English vocabulary acquisition, enhance students’ autonomous learning abilities, and foster effective learning strategies (Ling et al., 2019). These findings suggest that AI can provide significant support for university students’ learning within technology-enhanced learning environments and demonstrate notable advantages in facilitating multiple dimensions of their self-regulated learning (Zhu et al., 2025). As university students, pre-service teachers’ self-regulated learning may also be influenced by AI technological support. However, given their role as future educators, the relationship between AI technological support and pre-service teachers’ self-regulated learning is likely to be more profound and interconnected.

The use of AI technologies facilitates pre-service teachers’ self-regulated learning behaviours by promoting role transformation, enabling exposure to innovative teaching models, and supporting personalised reflective learning. First, the integration of AI technologies shifts the teacher’s role from knowledge transmitter to learning facilitator, thought inspirer, and innovation promoter. In AI-supported environments, pre-service teachers are better equipped to filter and evaluate information, engage in critical thinking, and stimulate intrinsic motivation for learning (Goyal, 2025). Second, by leveraging AI technological support, pre-service teachers, who are simultaneously learners and future educators, are able to experience and implement smart classroom models such as flipped classrooms, blended learning, and project-based learning (B. Q. Liu, 2020). This process supports the cultivation of their autonomous learning and lifelong learning awareness. At the same time, emerging technologies like AI provide pre-service teachers with rich, personalised intelligent learning resources alongside intuitive visual experiences, thereby enhancing their capacity for reflection, self-regulation, and self-monitoring (W. P. Hu et al., 2024). Ultimately, a key function of AI in enhancing pre-service teacher learning is to provide the data foundation for reflective teaching practice. By engaging with evidence-based feedback, pre-service teachers can refine their pedagogical understandings and develop adaptive instructional strategies (C. L. Wang et al., 2024), thereby fostering more effective self-regulated learning.

In terms of the relationship between school-based intelligent environment support and pre-service teachers’ self-regulated learning, school-based intelligent environment support, as an external support system, may be associated with pre-service teachers’ opportunities to engage in self-regulated learning. First, schools provide hardware resources that enable pre-service teachers to access and utilise AI technologies, supporting them in engaging in evidence-based learning planning, documentation, reflection, and regulation. Second, transformational leadership styles can enhance pre-service teachers’ motivation to engage in self-regulated learning through encouragement and support, while a school culture characterised by collaboration, openness, and respect for innovation provides social support for teachers’ reflective and innovative learning (Andiliou & Murphy, 2010). Furthermore, the departments and faculties can provide resources and reward mechanisms that encourage pre-service teachers to explore and implement new pedagogical approaches (Noviyanti et al., 2021). Accordingly, tangible AI resources and intangible forms of institutional support, including relevant documents, incentives, and policies, may be related to how pre-service teachers set goals, monitor progress, and regulate their learning processes.

In terms of the relationship between pre-service teachers’ self-regulated learning and their innovative competence, the essence of pre-service teachers’ instructional innovation is a process of creatively solving pedagogical problems within the context of teacher learning and teaching activities. Its core connotation involves pre-service teachers actively renewing their conceptions, adopting innovative teaching methods, creatively addressing instructional challenges, and fostering students’ own innovative development (X. Y. Yang & Shen, 2006). The very nature of pre-service teachers’ innovative competence encompasses characteristics associated with self-regulated learning, such as autonomy and reflexivity. The close relationship between pre-service teachers’ self-regulated learning and their innovative competence is well-documented, with existing evidence suggesting that self-regulated learning can facilitate the development of this competence. First, what distinguishes innovative teachers from their conventional counterparts is attributes such as innovation literacy, collaborative skills, risk-taking propensity, and visionary thinking (Keyhani & Kim, 2021). Pre-service teachers can develop and demonstrate these very attributes through self-regulated learning. For instance, by engaging in self-regulatory processes such as autonomous planning, self-monitoring of development, and dynamic self-assessment, pre-service teachers are able to reconstruct pedagogical approaches, assert their agency, and consequently enhance their instructional innovative competence (Jiang & Jiang, 2015). Second, research has found that students who use AI tools demonstrate significantly higher levels of critical thinking and autonomous learning ability compared to those who do not. Moreover, this enhancing effect of AI tools on critical thinking and autonomous learning is more pronounced among postgraduate students and those enrolled in “Double First-Class” universities than among undergraduate students and those in general higher education institutions (J. Qi et al., 2024). The underlying reason for this disparity is that the learning environments experienced by postgraduate and Double First-Class university students are characterised by a stronger culture of critical inquiry and autonomous learning, coupled with higher levels of self-regulatory learning willingness and proficiency. Within such contexts, AI technological support can more effectively amplify the development of innovative competence attributes like autonomous learning and critical thinking.

Furthermore, from the perspective of the intrinsic nature of self-regulated learning and the development of innovative competence, self-regulated learning profoundly activates learners’ multidimensional engagement. This includes the application of metacognitive strategies, the stimulation of intrinsic motivation, and the self-management of behaviour, thereby fully realising the potential of artificial intelligence to foster individual creativity. At the same time, through the proactive management and regulation of the learning process, self-regulated learning can reduce technological dependence and the motivation to plagiarise, thus effectively promoting students’ independent thinking and problem-solving abilities (du Rocher, 2020). Research has indicated that when university students’ interactions with AI remain superficial and lack engagement in self-regulated learning, AI not only fails to stimulate their creative potential but may instead become a hindrance to its development (S. Y. Wang & Huang, 2024). Therefore, self-regulated learning not only mitigates inappropriate use of AI among university students, including pre-service teachers, but also effectively reduces the negative impact of such misuse on their creativity.

Taken together, the literature on the relationship between AI technologies and learners’ innovative competence reveals two contrasting perspectives—namely, the facilitation perspective and the inhibition perspective (S. Y. Wang & Huang, 2024). The central point of contention in this debate concerns the manner in which learners engage with AI technologies. When individuals overcome technological dependence and proactively explore, connect, and reflect on learning materials, they can adjust, monitor, and evaluate their learning approaches and behaviors. Such engagement in self-regulated learning makes it more likely for AI technologies to promote their innovative competence. Building on research examining the relationships among AI technological support, school-based intelligent environment support, and pre-service teachers’ self-regulated learning, as well as the significant association between self-regulated learning and the development of innovative competence, it is plausible that self-regulated learning may play a mediating role in the relationships between AI technological support, school-based intelligent environment support, and pre-service teachers’ innovative competence. Studies investigating mediating and moderating processes in the relationship between AI and innovative work behaviour suggest that job crafting and humble leadership serve as important mediators and moderators, respectively, in the relationship between AI and employee innovative behaviour. Job crafting, which refers to the cognitive and behavioural adjustments employees make in response to the implementation of AI technologies, has been found to facilitate innovative behaviour (X. M. Li & Chen, 2024). Furthermore, learning goal orientation, as an individual-level moderating factor, can weaken the depletion pathway and strengthen the enrichment pathway of AI technology application, thereby influencing employees’ innovative behaviour (W. Zhang et al., 2024). Extending these insights to the teacher education context, self-regulated learning can be conceptualised as a form of “job crafting” for pre-service teachers, mediating the relationships between technological support, environmental factors, and the development of innovative competence.

### 2.4. Research Aims and Hypotheses

Based on the foregoing literature synthesis and theoretical excavation, this research aims to examine the associations of AI technological support and school-based intelligent environment support with pre-service teachers’ innovative competence, as well as the model-based indirect associations involving AI self-efficacy and self-regulated learning. AI self-efficacy and self-regulated learning were modelled as parallel mediators to compare their distinct indirect associations under the two forms of perceived support. Although the two constructs may be interrelated, social cognitive theory does not prescribe a unidirectional ordering between them, and the available evidence in AI-supported teacher education does not establish such an ordering. The parallel model therefore provides a parsimonious framework for examining differentiated mediation patterns, while serial mediation warrants examination in future longitudinal research. Accordingly, the following hypotheses were formulated.

**Hypothesis** **1.** *AI technological support is positively associated with pre-service teachers’ innovative competence.*

**Hypothesis** **2.** *School-based intelligent environment support is positively associated with pre-service teachers’ innovative competence.*

**Hypothesis** **3.** *AI self-efficacy mediates the relationship between AI technological support and pre-service teachers’ innovative competence.*

**Hypothesis** **4.** *AI self-efficacy mediates the relationship between school-based intelligent environment support and pre-service teachers’ innovative competence.*

**Hypothesis** **5.** *Self-regulated learning mediates the relationship between AI technological support and pre-service teachers’ innovative competence.*

**Hypothesis** **6.** *Self-regulated learning mediates the relationship between school-based intelligent environment support and pre-service teachers’ innovative competence.*

**Hypothesis** **7.** *The association of AI technological support with pre-service teachers’ innovative competence differs from that of school-based intelligent environment support. (That means the direct and indirect associations of AI technological support and school-based intelligent environment support with pre-service teachers’ innovative competence may differ. Moreover, the two types of support exhibit distinct patterns of association across the conditional distribution of innovative competence.)*

**Figure 1:**
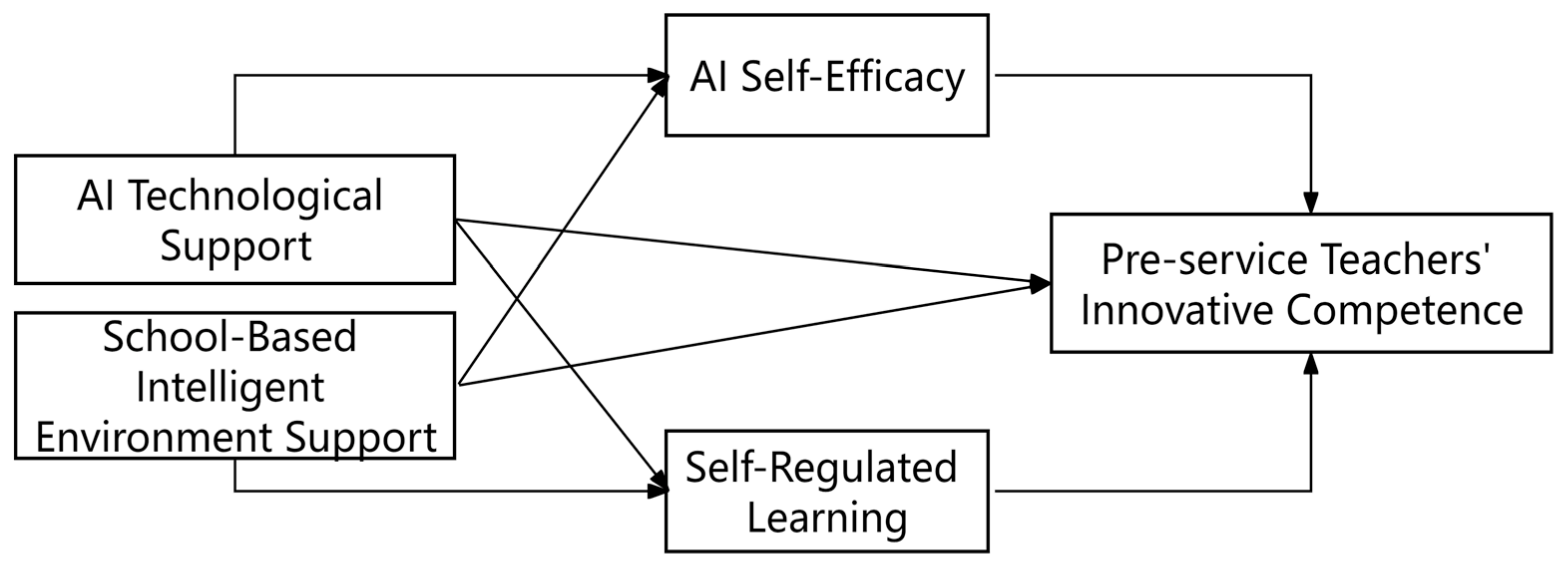
Theoretical model of the relationships among AI technological support, school-based intelligent environment support, AI self-efficacy, self-regulated learning, and pre-service teachers’ innovative competence.

Based on the above, a theoretical model depicting the mechanisms through which AI support is associated with pre-service teachers’ innovative competence is constructed, as illustrated in Figure 1.

## 3. Materials and Methods

### 3.1. Participants

The study focused on pre-service teachers at the undergraduate and professional master’s levels in higher education institutions across mainland China. A multi-site, institutionally facilitated convenience sampling approach was adopted. To increase the diversity of the sample across institutional and regional contexts, participants were recruited from higher education institutions located in 12 provinces in eastern, central, and western mainland China. The questionnaire was distributed through established institutional collaboration networks, with the assistance of principals, deans, teaching researchers, and faculty advisors from participating institutions. The questionnaire instructions emphasized principles including voluntariness, anonymity, and truthful completion; obtained informed consent from all participants; and ensured questionnaire validity through three measures: pre-survey control, in-survey monitoring, and post-survey screening. Specifically, first, we controlled the devices used for completing the electronic questionnaire, ensuring each device could only be used for one response. Second, through collaboration supported by the platform of the Center for Teacher Education Research at Beijing Normal University and the Teacher Education and Artificial Intelligence research team, we invited principals, deans, teaching researchers, and faculty advisors from relevant institutions to assist in distributing the electronic questionnaire and ensuring response quality. Third, we manually screened the returned questionnaires, excluding invalid ones with missing items or uniform responses such as fully compliant or very non-compliant. A total of 3214 questionnaires were collected, with 211 invalid ones excluded, leaving 3003 valid questionnaires and a valid response rate of 93.43%.

Because neither participating institutions nor individual respondents were selected through a probability-based sampling frame, this sample should not be interpreted as statistically representative of all pre-service teachers in China. Although the multi-province and multi-institutional recruitment strategy enhanced the diversity of the sample, the findings should be generalized cautiously, particularly to pre-service teachers in institutional and sociocultural contexts that differ from those represented in the present study. The basic information about the participants is shown in Table 1.

### 3.2. Variables and Measures

The study developed the questionnaire by referencing domestic and international research instruments and incorporating expert opinions. The empirical data for this study were collected exclusively in mainland China. The questionnaire was administered entirely in Chinese, and a rigorous translation and back-translation protocol was strictly adhered to prior to formal data collection to ensure linguistic accuracy and conceptual equivalence. The questionnaire comprised two sections. The first section collected basic information about pre-service teachers, including gender, grade level, and household registration, with categorical variables as the primary option types. The second section surveyed five variables: school-based intelligent environment support, AI technological support, AI self-efficacy, self-regulated learning, and pre-service teachers’ innovative competence. All five variables were rated using a 5-point Likert scale scoring method, with the scale ranging from 1 (Strongly Disagree) to 5 (Strongly Agree). To ensure the clarity and applicability of the questionnaire for the target population of pre-service teachers, all items were translated and back-translated in strict accordance with standard cross-cultural translation protocols to guarantee semantic equivalence. This questionnaire was adapted from multiple prior studies, drawing on the item wording and dimensions from relevant studies, with all referenced research instruments having been confirmed to possess high reliability and validity. Specifically, we implemented a formal item adaptation and validation process. A panel of six experts—comprising three specialists in educational technology, two in educational psychology, and one in teacher education—conducted a comprehensive review of the questionnaire. The experts evaluated each item across four dimensions: relevance, clarity, appropriateness, and alignment. Based on their iterative feedback, the wording of the items was refined to eliminate ambiguity. Consequently, all 31 items were carefully revised to fit the specific context and thematic focus of this study.

Specifically, first, the pre-service teachers’ innovative competence scale was adapted from B. M. Li et al.’s (2024) teacher innovation index system and G. X. Wu’s (2021) teacher innovation competence scale. It comprised three dimensions—Innovation Willingness, Innovation Readiness, and Innovation Practice—and included 9 items such as “I am willing to use AI to pioneer new teaching ideas.” Second, the self-regulated learning scale referenced the self-regulated learning scales of Winne and Perry (2000), S. Y. Wang and Huang (2024), and S. W.-Y. Lee and Tsai (2011), and included 5 items such as “I can monitor my progress and adjust learning content during the learning process.” Third, the AI self-efficacy scale referenced Rosen et al.’s (2013) Media and Technology Use and Attitude Scale and Xue’s (2015) Educational Technology Self-Efficacy Questionnaire and included 6 items such as “I believe AI technology can promote diversified instructional design.” Fourth, the AI technological support and school-based intelligent environment support scales primarily referenced the relevant scales of Chai et al. (2021), Venkatesh et al. (2003), Gou et al. (2024), and Teo (2010) and were developed through expert consultation into an assessment tool with 11 items, including “Using AI helps me simplify daily tasks” and “The intelligent education platform built by my school has rich learning resources.” The complete questionnaire, comprising all 31 measurement items with their corresponding original Chinese versions and English back-translations, is provided in Appendix A.

Because the support items asked participants to report their own evaluations and experiences, the measures capture perceived AI technological support and perceived school-based intelligent environment support rather than objectively verified technological provision or school-level environmental conditions.

### 3.3. Data Analysis

The study used IBM SPSS Amos (v26.0; IBM Corp., Armonk, NY, USA) and IBM SPSS Statistics (v27.0; IBM Corp., Armonk, NY, USA) as data analysis tools. The data analysis proceeded as follows. First, we used AMOS 26.0 to conduct a common method bias test among dimensions. Second, we used SPSS 27.0 and AMOS 26.0 to test the reliability and validity of the questionnaire. Third, we used SPSS 27.0 for descriptive and difference analyses and quantile regression analysis. Fourth, we used AMOS 26.0 to build a structural equation model, examine the theoretical relationships among variables, explore the direct and indirect associations of AI technological support and school-based intelligent environment support with pre-service teachers’ innovative competence, and adopt the bias-corrected percentile Bootstrap method to test the mediating effects of AI self-efficacy and self-regulated learning.

### 3.4. Common Method Bias Test

Common method bias is a systematic error that seriously misleads research results due to artificial covariation when using the questionnaire survey method. This error is influenced by factors such as data sources, questionnaire content and characteristics, and assessment environments, and it commonly exists in research fields like psychology (H. Zhou & Long, 2004). This study collected data via participant self-report, which may introduce common method bias. Thus, a common method bias test was necessary. We formed a single-factor model by combining five variables: school-based intelligent environment support, AI technological support, AI self-efficacy, self-regulated learning, and pre-service teachers’ innovative competence. The absolute fit indices [*χ*^2^/df = 78.119; RMR = 0.066; RMSEA = 0.160], comparative fit indices [IFI = 0.571, TLI = 0.541, CFI = 0.571], and parsimonious fit indices [PGFI = 0.385, PNFI = 0.530, PCFI = 0.533] of the single-factor model all failed to meet the fit criteria (Wen et al., 2004). The model showed poor fit with the actual data, and the fitting results were unsatisfactory. Simultaneously, we used the method of controlling for unmeasured latent method factors. After adding a latent common method factor to the five-factor model, the model goodness-of-fit did not improve significantly [ΔRMSEA = 0.010, ΔNFI = 0.020, ΔIFI = 0.021, ΔTLI = 0.022, ΔCFI = 0.021]. These results indicate that the survey data do not have serious common method bias issues.

### 3.5. Results of Questionnaire Reliability and Validity Tests

The results of the questionnaire reliability and validity tests are shown in Table 2 and Table 3. First, the overall Cronbach’s alpha coefficient of the questionnaire was 0.959, and the Cronbach’s alpha coefficients of the five dimensions all exceeded 0.90. Second, for construct validity, we conducted exploratory factor analysis using principal component analysis with varimax rotation. The Kaiser–Meyer–Olkin (KMO) measure of sampling adequacy was 0.960, and Bartlett’s test of sphericity yielded a significance level below 0.001. The orthogonally rotated component matrix converged after six iterations, extracting five common factors with a cumulative variance explained of 73.08%. The factor loadings of all items ranged from 0.60 to 0.85, indicating strong relationships between items and common factors and high homogeneity. The correspondence between items and common factors aligned with research expectations, demonstrating good construct validity of the questionnaire. Finally, confirmatory factor analysis results showed that all fit indices of the five-factor model [*χ*^2^/df = 14.240; RMR = 0.022; RMSEA = 0.066; NFI = 0.923; CFI = 0.928] met the fit criteria (L. Hu & Bentler, 1999; M. L. Wu, 2018), indicating a good fit between the five-factor model and the actual data. Additionally, the average variance extracted (AVE) of all five dimensions exceeded 0.50 (Hair et al., 2011), and the square roots of the AVE values for each dimension were greater than the correlation coefficients between paired dimensions, confirming good convergent and discriminant validity of the questionnaire data (Hair et al., 2011).

## 4. Results

### 4.1. Descriptive Statistics and Difference Analysis Results

Pre-service teachers’ innovative competence generally fell in the upper-middle range (*M* = 3.79, *SD* = 0.60). Among its subdimensions, innovation willingness (*M* = 3.87, *SD* = 0.62) and innovation readiness (*M* = 3.83, *SD* = 0.64) were relatively higher, while innovation practice (*M* = 3.68, *SD* = 0.72) was relatively lower. Difference analysis results showed no significant differences in pre-service teachers’ innovative competence by gender or institution type. However, pre-service teachers with urban household registration [*t* (3001) = −2.342, *p* = 0.019, *Cohen’s d* = 0.086], humanities majors [*F* (3, 2999) = 5.638, *p* < 0.001, *η*^2^ = 0.006], above-undergraduate grade levels [*F* (2, 3000) = 9.298, *p* < 0.001, *η*^2^ = 0.006], annual family incomes over 50,000 yuan [*F* (2, 3000) = 8.662, *p* < 0.001, *η*^2^ = 0.006], parents with junior college or bachelor’s degrees [*F* (2, 3000) = 6.056, *p* = 0.002, *η*^2^ = 0.004], medium-to-high AI usage frequencies [*F* (2, 3000) = 44.017, *p* < 0.001, *η*^2^ = 0.029], and medium-to-high AI interest levels [*F* (2, 3000) = 197.276, *p* < 0.001, *η*^2^ = 0.116] exhibited significantly higher overall innovative competence.

For differences in the three subdimensions of innovative competence: First, pre-service teachers with urban household registration [*t* (3001) = −2.450, *p* = 0.014, *Cohen’s d* = 0.089], comprehensive institutions [*t* (3001) = 2.234, *p* = 0.026, *Cohen’s d* = 0.103], humanities majors [*F* (3, 2999) = 7.947, *p* < 0.001, *η*^2^ = 0.008], above-undergraduate grade levels [*F* (2, 3000) = 18.188, *p* < 0.001, *η*^2^ = 0.012], annual family incomes over 50,000 yuan [*F* (2, 3000) = 13.152, *p* < 0.001, *η*^2^ = 0.009], parents with junior college or bachelor’s degrees [*F* (2, 3000) = 4.922, *p* = 0.007, *η*^2^ = 0.003], medium-to-high AI usage frequencies [*F* (2, 3000) = 38.097, *p* < 0.001, *η*^2^ = 0.025], and medium-to-high AI interest levels [*F* (2, 3000) = 169.419, *p* < 0.001, *η*^2^ = 0.101] showed significantly higher innovation willingness.

Second, those with urban household registration [*t* (3001) = −2.524, *p* = 0.012, *Cohen’s d* = 0.092], humanities majors [*F* (3, 2999) = 7.996, *p* < 0.001, *η*^2^ = 0.008], above-undergraduate grade levels [*F* (2, 3000) = 11.87, *p* < 0.001, *η*^2^ = 0.008], annual family incomes over 50,000 yuan [*F* (2, 3000) = 9.247, *p* < 0.001, *η*^2^ = 0.006], parents with junior college or bachelor’s degrees [*F* (2, 3000) = 4.379, *p* = 0.013, *η*^2^ = 0.003], medium-to-high AI usage frequencies [*F* (2, 3000) = 35.636, *p* < 0.001, *η*^2^ = 0.023], and medium-to-high AI interest levels [*F* (2, 3000) = 190.355, *p* < 0.001, *η*^2^ = 0.113] had significantly higher innovation readiness.

Finally, male pre-service teachers [*t* (3001) = 2.313, *p* = 0.021, *Cohen’s d* = 0.112], normal universities [*t* (3001) = −1.986, *p* = 0.047, *Cohen’s d* = 0.095], parents with junior college or bachelor’s degrees [*F* (2, 3000) = 5.595, *p* = 0.004, *η*^2^ = 0.004], medium-to-high AI usage frequencies [*F* (2, 3000) = 34.255, *p* < 0.001, *η*^2^ = 0.022], and medium-to-high AI interest levels [*F* (2, 3000) = 124.143, *p* < 0.001, *η*^2^ = 0.076] exhibited significantly higher innovation practices. Overall, pre-service teachers’ innovative competence and its three subdimensions showed similar trends of heterogeneity across different background variables. The effect sizes (eta-squared, *η*^2^) for differences in AI usage frequency all exceeded the low-effect criterion of 0.01, and those for AI interest level all exceeded the medium-effect criterion of 0.06. This indicates that AI interest level has a stronger association with pre-service teachers’ innovative competence than AI usage frequency.

### 4.2. Quantile Regression Analysis Results

We used a quantile regression model to explore whether the estimated associations of AI technological support and school-based intelligent environment support with pre-service teachers’ innovative competence differ across different quantile levels. We set quantile points from 0.01 to 0.90, connected the estimated coefficients at each quantile point to reflect the overall impact trend, and the results are shown in Table 4. Results showed that both AI technological support and school-based intelligent environment support were positively associated with pre-service teachers’ innovative competence at all quantile levels, but the magnitude and pattern of these estimated associations differed. First, the estimated association of AI technological support with innovative competence generally followed a rising wave-like pattern across the middle-to-upper quantiles, with stronger estimated associations at medium-to-high levels of innovative competence. Second, the estimated association of school-based intelligent environment support with innovative competence followed an inverted U-shaped pattern, with stronger estimated associations at medium-to-low levels of innovative competence. This pattern does not establish a ceiling effect or diminishing marginal returns caused by school-based intelligent environment support; rather, it indicates that the estimated cross-sectional association varied across quantiles of innovative competence. These distinct quantile patterns are consistent with Hypothesis 7, indicating that the estimated associations of AI technological support and school-based intelligent environment support with pre-service teachers’ innovative competence differed in magnitude and pattern across the conditional distribution of innovative competence.

### 4.3. Structural Equation Model Test Results

**Figure 2:**
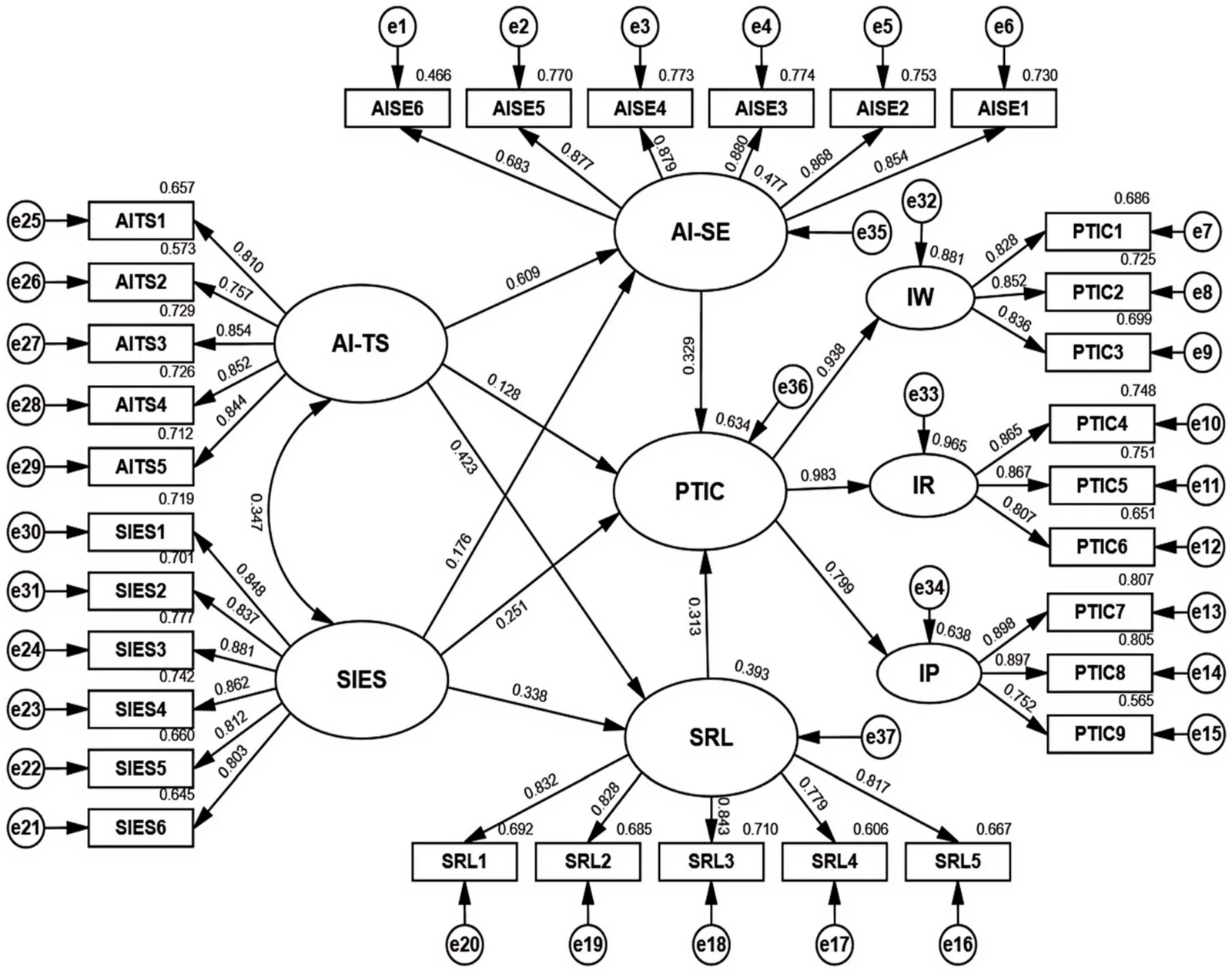
Structural model of the relationships among AI technological support (AI-TS), school-based intelligent environment support (SIES), AI self-efficacy (AI-SE), self-regulated learning (SRL), and pre-service teachers’ innovative competence (PTIC). Note: innovation willingness = IW; innovation readiness = IR; innovation practice = IP.

To explore the “dual-drive dual-mediation” model of associations with pre-service teachers’ innovative competence, the study constructed a structural equation model and used the maximum likelihood method for parameter estimation. The results are shown in Figure 2. All model fit indices [*χ*^2^/df = 9.624; RMR = 0.026; RMSEA = 0.054; GFI = 0.915; CFI = 0.953] met the fit criteria, indicating a good overall model fit (Wen et al., 2004). Path results are shown in Table 5. AI technological support had standardized path coefficients of 0.128, 0.609, and 0.423 for pre-service teachers’ innovative competence, AI self-efficacy, and self-regulated learning, respectively. School-based intelligent environment support had standardized path coefficients of 0.251, 0.176, and 0.338 for pre-service teachers’ innovative competence, AI self-efficacy, and self-regulated learning, respectively. AI self-efficacy had a standardized path coefficient of 0.329 for pre-service teachers’ innovative competence, and self-regulated learning had a standardized path coefficient of 0.313 for pre-service teachers’ innovative competence. All path significance levels were less than 0.001. In summary, hypotheses 1 and 2 were supported. This indicates that school-based intelligent environment support and AI technological support had significant positive associations with pre-service teachers’ AI self-efficacy, self-regulated learning, and innovative competence. Both AI self-efficacy and self-regulated learning also had direct significant positive associations with their innovative competence. The pronounced difference in the direct association magnitudes between the two types of support further corroborated Hypothesis 7.

### 4.4. Mediating Effect Test Results

The study explored the model-based indirect associations involving AI self-efficacy and self-regulated learning, linking AI technological support and school-based intelligent environment support with pre-service teachers’ innovative competence using the stepwise testing method. It also employed the Bootstrap method with 5000 bootstrapping samples to estimate the confidence intervals for the mediating effects. Results are shown in Table 6. A mediating effect is considered significant if the 95% Bootstrap confidence interval for the mediating effect does not include 0.

Among the two mediating paths of AI technological support associated with pre-service teachers’ innovative competence, first, the total effect of AI-TS→AI-SE→PTIC was 0.294 and significant (confidence interval [0.246, 0.345], *p* < 0.001). The direct effect of AI-TS→PTIC was 0.115 and significant (confidence interval [0.069, 0.162], *p* < 0.001). The mediating effect of AI-TS→AI-SE→PTIC was 0.179 and significant (confidence interval [0.147, 0.213], *p* < 0.001). This indicates that AI self-efficacy plays a partial mediating role in the path from AI technological support to pre-service teachers’ innovative competence, accounting for 60.88% of the total effect, validating hypothesis 3. Second, the total effect of AI-TS→SRL→PTIC was 0.233 and significant (confidence interval [0.185, 0.284], *p* < 0.001). The mediating effect of AI-TS→SRL→PTIC was 0.119 and significant (confidence interval [0.092, 0.147], *p* < 0.001). This indicates that self-regulated learning also plays a partial mediating role in this path, accounting for 51.07% of the total effect, validating hypothesis 5. Comparing the mediating effects, the point estimate was 0.061. The Bias-corrected and 95% percentile confidence intervals did not include 0, indicating that the mediating effect of AI self-efficacy was significantly greater than that of self-regulated learning.

Among the two mediating paths, school-based intelligent environment support was associated with pre-service teachers’ innovative competence. First, the total effect of SIES→AI-SE→PTIC was 0.242 and significant (confidence interval [0.203, 0.284], *p* < 0.001). The direct effect of SIES→PTIC was 0.197 and significant (confidence interval [0.161, 0.235], *p* < 0.001). The indirect effect of SIES→AI-SE→PTIC was 0.045 and significant (confidence interval [0.031, 0.062], *p* < 0.001). This indicates that AI self-efficacy plays a partial mediating role in the path from school-based intelligent environment support to pre-service teachers’ innovative competence, accounting for 18.60% of the total effect, validating hypothesis 4. Second, the total effect of SIES→SRL→PTIC was 0.280 and significant (confidence interval [0.241, 0.320], *p* < 0.001). The indirect effect of SIES→SRL→PTIC was 0.083 and significant (confidence interval [0.062, 0.106], *p* < 0.001). This indicates that self-regulated learning also plays a partial mediating role in this path, accounting for 29.64% of the total effect, validating hypothesis 6. Comparing the mediating effects, the point estimate was −0.038. The bias-corrected and 95th percentile confidence intervals did not include 0, indicating that the mediating effect of AI self-efficacy was significantly lower than that of self-regulated learning. This pattern further supports Hypothesis 7 by indicating that the two supports showed different model-based indirect association patterns with innovative competence.

## 5. Discussion

As shown in Table 7, the estimated associations were consistent with all seven hypotheses proposed in this study. The positive associations of AI technological support (H1) and school-based intelligent environment support (H2) with innovative competence were observed, and AI self-efficacy (H3 and H4) and self-regulated learning (H5 and H6) showed statistically significant model-based indirect associations linking the two support variables with innovative competence. Hypothesis 7 was supported by the quantile-regression results and structural equation modeling, which indicated that the estimated associations of the two types of support with innovative competence differed in magnitude and pattern across the conditional distribution of innovative competence. Specifically, AI technological support exhibited a gradually rising wave-like pattern across increasing quantiles of innovative competence, with stronger estimated associations at medium-to-high levels of innovative competence. In contrast, school-based intelligent environment support displayed an inverted U-shaped pattern of estimated associations, with stronger estimated associations at medium-to-low levels of innovative competence and weaker estimated associations at higher quantiles. These heterogeneous patterns are consistent with the proposed theoretical framework and indicate different statistical patterns of association linking the two forms of support with innovative competence.

### 5.1. Differences in Pre-Service Teachers’ Innovative Competence Across Individual, Family, and School Multi-Level Contextual Variables

This study identified significant differences in pre-service teachers’ innovative competence across three categories of background variables: individual, family, and school. With respect to individual-level variables, several patterns emerged. First, male pre-service teachers demonstrated slightly higher levels of innovative competence than their female counterparts, although this difference did not reach statistical significance. While some previous studies have reported a significant positive effect of gender on pre-service teachers’ innovative competence (Chen et al., 2024; W. P. Hu & Han, 2015), others have found no significant gender differences in student teachers’ innovative spirit (Mao et al., 2019). The present findings corroborate the convergence of innovative competence between genders among pre-service teachers in the AI era. Second, pre-service teachers from urban household registrations exhibited significantly higher levels of innovative competence than those from rural registrations. This finding reflects the persistent imbalance and disparity in educational resource allocation between urban and rural areas in China (W. Li et al., 2024). It is plausible that urban pre-service teachers, through earlier exposure to science and innovation competitions and platforms, have greater opportunities to shape their innovative thinking and practical skills, thereby demonstrating stronger innovative efficacy. Third, pre-service teachers with a higher frequency of AI use demonstrated higher levels of innovative competence. Teacher innovative competence is central to the integration of AI in education, with particular emphasis on cultivating AI-based innovation awareness and creativity (Y. Y. Yang et al., 2022). Research has indicated that AI use significantly enhances postgraduate students’ research innovation competence (Ma et al., 2024), suggesting that frequent engagement with AI technologies among pre-service teachers helps stimulate their innovative potential and enhance their innovative competence through human–computer interaction. However, caution is warranted regarding the potential reinforcement of instrumental thinking through AI automation (G. Zhou, 2025) and the risk of cognitive outsourcing associated with the application of intelligent technologies in education (S. Q. Yu & Wang, 2023). These concerns may subtly erode pre-service teachers’ professional agency and autonomy by displacing pedagogical decision-making to algorithmic prescriptions, thereby reducing teaching to mere procedural execution. Simultaneously, the extensive data collection embedded in AI-driven platforms raises significant data privacy concerns, as sensitive behavioural and performance information becomes vulnerable to opaque processing. Addressing these challenges thus necessitates the cultivation of critical AI literacy, equipping pre-service teachers with the ethical discernment to interrogate algorithmic outputs, safeguard data integrity, and preserve the human-centred core of their professional practice. Fourth, pre-service teachers with greater interest in AI also exhibited higher levels of innovative competence. As the intrinsic driver of educational innovation, interest-driven AI application is more likely to foster sustained interaction and collaborative innovation.

Regarding family background, two key findings warrant attention. On one hand, pre-service teachers from families with higher annual incomes demonstrated higher levels of innovative competence. Extensive research has established a positive correlation between family socioeconomic status and students’ creative development (Castillo-Vergara et al., 2018; Miller & Gerard, 1979). Higher-income families are better positioned to support innovation-related decisions and provide platforms for innovative practice and technological engagement, thereby effectively expanding pre-service teachers’ cognitive boundaries of innovation and facilitating the development of systematic problem-solving strategies for innovation. On the other hand, pre-service teachers whose parents held a bachelor’s or associate degree as their highest level of education also exhibited higher levels of innovative competence. Parental education level, as a core component of family cultural capital, is associated with more systematic disciplinary thinking and a greater emphasis on cultivating innovative competence and exploratory spirit (W. H. Huang & Huang, 2024), thereby fostering pre-service teachers’ innovative thinking from an early stage.

Turning to school-level factors, institutional type and disciplinary background revealed notable patterns. First, pre-service teachers from comprehensive universities demonstrated slightly higher levels of innovative competence than those from teacher education universities, although this difference was not statistically significant. A possible explanation is that the interdisciplinary environmental support and diverse systematic training characteristic of comprehensive universities have not yet fully manifested their influence on pre-service teachers. The training focus for pre-service teachers in both comprehensive and teacher education universities remains predominantly on pedagogical theory and instructional skills. Second, pre-service teachers specialising in humanities and social sciences exhibited the highest levels of innovative competence. This finding diverges from previous research indicating that science and mathematics teachers demonstrate significantly higher innovative competence than language teachers (G. X. Wu, 2021). A possible explanation is that humanities and social sciences pre-service teachers, through philosophical reasoning and related approaches, are better equipped to integrate humanistic rationality into educational and pedagogical innovation. They demonstrate particular innovative potential in incorporating humanities and social science paradigms into ethical considerations surrounding AI in education. Third, pre-service teachers in upper-level undergraduate programmes demonstrated the highest levels of innovative competence. This finding contrasts with previous research reporting no significant grade-level differences in normal students’ innovative spirit (Mao et al., 2019). The discrepancy may be attributed to the richer teaching experience accumulated by upper-level pre-service teachers through a process of “practice–reflection–re-practice,” which enables them to solve problems creatively through knowledge transformation and, in turn, enhance their innovative competence.

### 5.2. Direct Associations of AI Technological Support and School-Based Intelligent Environment Support with Pre-Service Teachers’ Innovative Competence

This study found that both AI technological support and school-based intelligent environment support were significantly and positively associated with pre-service teachers’ innovative competence, supporting Hypotheses 1 and 2. Their estimated associations followed distinct patterns across quantiles of innovative competence: AI technological support exhibited a “rising, wave-like pattern,” whereas school-based intelligent environment support demonstrated an inverted U-shaped trend. In the specified model, school-based intelligent environment support had a larger direct path estimate than AI technological support. These differences in estimated association patterns and path estimates were consistent with Hypothesis 7.

On one hand, the deep integration of AI technologies with teacher innovation has the potential to fundamentally transform the generation of pedagogical innovations. By providing intelligent tools, interactive platforms, and virtual contexts, AI can facilitate the activation of pre-service teachers’ innovative inspiration. Drawing on technology acceptance models, research suggests that when teachers receive adequate support and assurance regarding AI use, they are more likely to develop positive technology perceptions and experience reduced AI-related anxiety (Koedinger & Corbett, 2006), thereby effectively promoting innovative development in their teaching practice. Regarding the evaluation of innovative competence, AI-powered systems can continuously refine assessment mechanisms, enabling innovators to reflect upon, validate, and evaluate their own innovative activities and to dynamically adjust these activities and highlight innovative outcomes based on evaluative feedback (Gao & Yan, 2024). Thus, AI’s significant potential in the innovation domain offers new pathways for developing students’ innovative competence (J. J. Zhang & Gao, 2020). The rising, wave-like pattern of AI technological support indicates stronger estimated associations among pre-service teachers with moderate to high levels of innovative competence. This suggests that the efficacy of AI technological support depends, to a considerable extent, on pre-service teachers’ existing innovation willingness, readiness, and practice. As AI becomes increasingly integrated with education, it not only supports teachers’ pedagogical innovation in areas such as personalised instruction, professional development, and home-school collaboration but also places higher demands on their innovative competence (He & Guo, 2021). However, the estimated associations of AI technological support were not uniform across levels of innovative competence; rather, they were hierarchical, conditional, and differentiated. How to leverage emerging technologies such as AI to create integrated and diversified learning environments will be critical to transforming pre-service teacher education models.

On the other hand, schools can exert a more substantial influence on pre-service teachers’ innovative competence by constructing a multi-dimensional intelligent support environment. Within such environments, pre-service teachers can enhance their innovative competence through activities such as forming communities of innovative practice. Previous research has established a significant association between school environmental support and student teachers’ innovation, demonstrating that peer influence, university curricula, and teacher support collectively and positively predict normal students’ innovative spirit (Mao et al., 2019). However, this study found stronger estimated associations of school-based intelligent environment support among pre-service teachers with low to moderate levels of innovative competence. This pattern indicates variation in the estimated cross-sectional association across quantiles and does not demonstrate a ceiling effect or diminishing marginal returns. At a critical juncture when teacher education is undergoing profound transformation in its goals, curricula, models, and assessment, AI is bringing fundamental changes to both the theoretical learning and teaching practice of pre-service teachers (M. Lin et al., 2024). Schools can positively influence pre-service teachers’ innovative competence through diverse forms of support, including offering AI-related courses, cultivating intelligent education faculty, organising AI competitions, constructing intelligent support platforms, providing dedicated funding, and developing AI-related policies. Furthermore, AI-driven intelligent learning environments and assessment systems can facilitate students’ immersive learning and innovative exploration (Xu & Shen, 2023), effectively reducing their perception of risk when engaging in educational innovation and experimental practice, thereby strengthening the cultivation of innovative competence through the activation of their own innovative potential.

In summary, AI technological support and school-based intelligent environment support are fundamentally distinct in nature. The former is primarily associated with innovative competence at the cognitive level, whereas the latter represents the sociocultural context in which innovative competence is developed. Although the two forms of support exhibit different overall trends in their effects across quantiles of pre-service teachers’ innovative competence, together they constitute an integrated “technology-embedded and environment-reconstructed” intelligent support system. This integrated system holds significant potential for facilitating pre-service teachers’ engagement in innovative activities and enhancing their innovative competence.

### 5.3. Partial Mediating Role of AI Self-Efficacy Between AI Support and Pre-Service Teachers’ Innovative Competence

The mediation analysis showed statistically significant model-based indirect associations involving AI self-efficacy in the relationships of AI technological support and school-based intelligent environment support with innovative competence, supporting Hypotheses 3 and 4.

On one hand, AI technological support was positively associated with pre-service teachers’ AI self-efficacy, and AI self-efficacy was positively associated with innovation willingness and innovation readiness during professional learning. However, caution is warranted: the instrumental transmission of AI technological support via self-efficacy, while efficient, may risk fostering an over-reliance on algorithmic guidance, thereby subtly eroding pre-service teachers’ professional agency and encouraging cognitive outsourcing if critical reflection on AI outputs is absent. Previous research has demonstrated that the frequency of AI use significantly and positively influences students’ AI self-efficacy (L. Zhang & Xu, 2025), suggesting that when AI empowers pre-service teachers by reinforcing their competence beliefs, a “technology—efficacy—innovation” chain reaction occurs. That is, the model indicated a statistically significant indirect association linking AI technological support with innovative competence via AI self-efficacy. To prevent this chain reaction from devolving into passive instrumentality, the cultivation of robust AI literacy is imperative—equipping pre-service teachers not only with technical proficiency but also with the ethical discernment to interrogate AI-generated suggestions and safeguard their own pedagogical autonomy.

On the other hand, school-based intelligent environment support was positively associated with pre-service teachers’ AI self-efficacy. Intelligent learning systems, evaluation systems, collaborative innovation mechanisms, and adaptive support mechanisms are all underpinned by AI. These systems can effectively enhance pre-service teachers’ understanding and mastery of AI, thereby strengthening their competence beliefs and perceptions of AI use. This heightened competence belief, in turn, leads to increased innovation willingness and innovation readiness. Notably, the two mediating pathways differed in their relative contribution. The pathway through AI technological support accounted for a higher proportion of the mediated effect than did the pathway through school-based intelligent environment support. This difference in indirect-association magnitude was consistent with Hypothesis 7. It should be interpreted as a difference in model-based statistical pathways rather than evidence of distinct causal psychological mechanisms.

Together, both model-based indirect associations were consistent with a statistical pattern linking AI-related support with innovative competence via internal efficacy beliefs.

### 5.4. Partial Mediating Role of Self-Regulated Learning Between AI Support and Pre-Service Teachers’ Innovative Competence

The present study found that pre-service teachers’ self-regulated learning partially mediated the relationship between AI technological support and their innovative competence, as well as the relationship between school-based intelligent environment support and their innovative competence, thereby providing full empirical support for Hypothesis 5 and Hypothesis 6.

Self-regulated learning is conceptualized as a cyclical and dynamic learning process encompassing four key phases: planning, monitoring, adjustment, and evaluation (Zimmerman, 1989). It involves learners regulating their cognition, motivation, emotions, behavior, and context, all systematically oriented toward the achievement of learning goals (Pintrich, 2000). AI technologies can facilitate the construction of a closed-loop system for self-regulated learning by providing real-time evaluation, intelligent diagnosis, dynamic feedback, and personalized adaptation, thereby offering pre-service teachers dynamically optimized learning models. However, the very efficiency of such AI-driven self-regulation support carries an inherent risk: when AI systems preemptively structure learning pathways and offer ready-made regulatory prompts, pre-service teachers may inadvertently outsource core metacognitive functions, compromising the cultivation of genuine self-regulatory competence and diminishing their professional autonomy in directing their own learning trajectories. Moreover, research has demonstrated that AI support exerts a significant positive influence on self-regulated learning capacity, while self-regulated learning itself can mitigate the negative effects of inappropriate AI use (S. Y. Wang & Huang, 2024). In the present study, the model-based indirect association involving self-regulated learning was consistent with the proposed statistical pathway linking AI-related support with innovative competence. It is worth noting, however, that the effectiveness of this pathway depends fundamentally on pre-service teachers’ own innovative awareness and mindset, underscoring the importance of their proactive integration of intelligent technologies into educational innovation.

Self-regulated learning also functions as a key nexus connecting school-based intelligent environment support with pre-service teachers’ innovative competence. Through initiatives such as virtual internship classrooms, intelligent training platforms, AI-enhanced curriculum development, and the cultivation of AI-literate educators, schools are progressively constructing a systematic intelligent support environment. This environment provides contextualized support that is particularly conducive to fostering pre-service teachers’ self-regulated learning. Furthermore, tangible hardware resources and intangible support, such as AI-related policies and incentives, may be associated with self-regulated learning capacity. In the specified model, self-regulated learning showed a statistically significant indirect association linking school-based intelligent environment support with innovative competence. Yet, as these environments become increasingly data-intensive, the systematic logging of self-regulatory behaviours, including planning patterns, monitoring frequencies, and help-seeking actions, poses significant data privacy challenges. Without robust ethical safeguards and transparent data stewardship, the very infrastructure designed to foster self-regulation may inadvertently subject pre-service teachers to intrusive surveillance, undermining the trust essential for authentic professional growth.

A notable difference emerged between the two mediating pathways. The indirect effect of AI technological support through self-regulated learning was proportionally larger than that of school-based intelligent environment support. This difference in model-based indirect associations was consistent with Hypothesis 7, which posited different estimated association patterns for the two types of support. In the specified model, the indirect association involving AI technological support and self-regulated learning was larger than the corresponding association involving school-based intelligent environment support.

### 5.5. Limitations and Future Research

Although this study drew on a sample of 3003 pre-service teachers from 12 provinces in mainland China and employed quantile regression and structural equation modeling to systematically examine the associations among AI technological support, intelligent environmental support in schools, AI self-efficacy, self-regulated learning, and pre-service teachers’ innovative competence, several limitations should be acknowledged.

From a temporal and causal-inference perspective, this study adopted a cross-sectional design, with all five core variables measured during the same survey period. Accordingly, the findings identify patterns of associations among AI-related support, AI self-efficacy, self-regulated learning, and pre-service teachers’ innovative competence, as well as model-based indirect associations consistent with the proposed theoretical model. However, neither the temporal ordering nor the causal relationships among these variables can be established. For example, pre-service teachers with higher innovative competence may be more likely to actively seek, access, and use AI technological support. This limitation also bears on the mediation specification. Although AI self-efficacy and self-regulated learning were modelled as parallel mediators to estimate their distinct indirect associations, their relationship may also follow a serial or reciprocal pattern. Accordingly, future research should employ longitudinal designs with multiple measurement waves and temporally separated measures to examine dynamic relations among AI-related support, individual psychological beliefs, learning processes, and innovative competence. Such designs would allow direct comparisons of parallel and serial mediation models and provide clearer evidence regarding the temporal ordering and potential reciprocal association between AI self-efficacy and self-regulated learning. In addition, experimental or quasi-experimental designs could complement longitudinal research by comparing the differentiated effects of AI-support interventions on the development of pre-service teachers’ innovative competence.

With respect to data sources and measurement, this study relied primarily on self-reported questionnaire data from pre-service teachers. Although the single-factor model comparison and the unmeasured latent method factor approach did not indicate serious common method bias, data collected from the same source, through the same measurement method, and within the same survey period may still be influenced by social desirability, pre-service teachers’ self-perceptions, and common-source bias. These factors may lead to the overestimation or underestimation of the associations among the variables. Future research could integrate questionnaire responses with multiple sources of evidence, including AI-platform usage records, learning-process data, classroom observations, instructional design artifacts, and evaluations from university teachers or peers. Such triangulation would enable a more comprehensive assessment of the development of pre-service teachers’ innovation willingness, innovation readiness, and innovation practice in AI-supported contexts.

Regarding sampling and the scope of generalizability, the multi-site recruitment strategy across 12 provinces in eastern, central, and western mainland China enabled the study to obtain a sample with a degree of regional and institutional diversity. However, the questionnaire was distributed primarily through a collaborative institutional network coordinated by the Center for Teacher Education Research at Beijing Normal University, with the assistance of relevant administrators, faculty members, and researchers. The sample was therefore not obtained through probability sampling based on a national sampling frame of pre-service teachers. Accordingly, it should not be regarded as statistically representative of all pre-service teachers in China, and the applicability of the findings across different countries, cultural contexts, teacher education systems, and institutional settings requires further examination. Future studies could employ stratified, multistage probability sampling to broaden sample coverage and conduct comparative research across regions, institutional types, and cultural contexts to further assess the robustness and external validity of the present findings.

At the construct-operationalization level, this study operationalized AI technological support as pre-service teachers’ overall perceptions of the support provided by AI technologies during learning and teaching preparation. The measure primarily captured their evaluations of the value of AI use, efficiency enhancement, task support, and learning potential. This operationalization facilitated an overall examination of the relationship between AI technological support and innovative competence. However, it did not distinguish among different forms of AI support, such as generative AI, intelligent tutoring systems, intelligent feedback tools, and intelligent simulation environments. Nor did it fully capture the distinctive characteristics of different AI tools in terms of frequency of use, pedagogical purposes, modes of human-AI collaboration, and ethical risks. Future research could develop a typological and multidimensional measurement framework for AI technological support, compare the specific mechanisms associated with different forms of AI support, and incorporate factors such as AI ethics awareness, algorithmic trust, critical judgment, and the quality of human-AI collaboration into the analysis. Such efforts would deepen understanding of the boundaries and conditions under which AI can support the development of pre-service teachers’ innovative competence.

Overall, these limitations indicate that future research should employ designs with stronger temporal evidence, behavioral data, more diverse samples, and more refined measurements to further examine the patterns of associations and potential mechanisms proposed in this study.

## 6. Conclusions

Using cross-sectional survey data from 3003 pre-service teachers in mainland China, this study examined the associations among AI technological support, school-based intelligent environment support, AI self-efficacy, self-regulated learning, and pre-service teachers’ innovative competence. The findings indicated significant differences in innovative competence across individual, family, and school-level background variables. Notably, urban or rural registration, frequency of AI use, interest in AI, family income, parental education, academic discipline, and grade level showed significant associations with innovative competence. Both AI technological support and school-based intelligent environment support were significantly and positively associated with innovative competence. School-based intelligent environment support showed a larger standardized path estimate in the specified model, and its estimated association with innovative competence followed an inverted U-shaped pattern across the examined quantiles. In contrast, the association of AI technological support generally followed a rising wave-like pattern across the middle-to-upper quantiles. These patterns describe heterogeneity in the estimated cross-sectional associations. Additionally, AI self-efficacy and self-regulated learning showed statistically significant model-based indirect associations linking both forms of support with innovative competence. This study provides correlational evidence relevant to understanding the associations among AI-related support, psychological factors, learning processes, and innovative competence in pre-service teacher education. It also offers insights relevant to the design of initiatives intended to support pre-service teachers’ innovative competence.

## Figures and Tables

**Table 1:** Demographic Characteristics of Study Participants.

Variables	Categories	Number	Percentage/%
Gender	Male	591	19.68
	Female	2412	80.32
Household Registration	Rural	1549	51.58
	Urban	1454	48.42
Major Type	Humanities and Social Sciences	622	20.71
	Science and Engineering	1021	34.00
	Arts and Sports	308	10.26
	Comprehensive	1052	35.03
Institution Type	Comprehensive	534	17.78
	Normal	2469	82.22
Identity Type	Publicly funded Teacher Education Students	427	14.22
	Non-publicly funded Teacher Education Students	2576	85.78
Grade Level	Lower undergraduate grades	1107	36.86
	Upper undergraduate grades	960	31.97
	Above undergraduate level	936	31.17
AI Usage Frequency	Low frequency (0–5 times/week)	1530	50.95
	Medium frequency (6–10 times/week)	962	32.03
	High frequency (≥11 times/week)	511	17.02
AI Interest Level	No interest	45	1.50
	Little interest	105	3.50
	Neutral	946	31.50
	Moderately interested	1203	40.06
	Very interested	704	23.44
AI Usage Contexts (Multiple Choice)	Course learning	2660	88.58
	Research activities	1233	41.06
	Daily life	1927	64.17
	Further education and job hunting	624	20.78
	Others	168	5.59

**Table 2:** Reliability and Convergent Validity Test.

Variable	Items	Standardized Factor Loadings	Parameter Significance Estimate			AVE	CR	Cronbach’s α
			*SE*	*t-Value*	*p*			
SIES	SIES1	0.85				0.706	0.935	0.935
	SIES2	0.84	0.017	58.007	***			
	SIES3	0.88	0.016	63.367	***			
	SIES4	0.86	0.016	60.841	***			
	SIES5	0.81	0.018	55.203	***			
	SIES6	0.80	0.017	54.134	***			
AI-TS	AITS1	0.81				0.684	0.915	0.913
	AITS2	0.76	0.022	46.140	***			
	AITS3	0.86	0.019	54.485	***			
	AITS4	0.85	0.019	54.319	***			
	AITS5	0.85	0.020	53.546	***			
AI-SE	AISE1	0.85				0.711	0.936	0.933
	AISE2	0.87	0.016	63.078	***			
	AISE3	0.88	0.016	64.685	***			
	AISE4	0.88	0.016	64.493	***			
	AISE5	0.88	0.016	64.310	***			
	AISE6	0.68	0.020	43.185	***			
SRL	SRL1	0.83				0.673	0.911	0.911
	SRL2	0.83	0.019	53.815	***			
	SRL3	0.84	0.019	55.310	***			
	SRL4	0.78	0.020	49.258	***			
	SRL5	0.82	0.018	52.927	***			
PTIC	PTIC1	0.78				0.627	0.938	0.936
	PTIC2	0.80	0.022	48.017	***			
	PTIC3	0.82	0.023	49.598	***			
	PTIC4	0.84	0.023	51.525	***			
	PTIC5	0.85	0.022	51.917	***			
	PTIC6	0.82	0.022	49.754	***			
	PTIC7	0.76	0.025	45.052	***			
	PTIC8	0.77	0.025	45.636	***			
	PTIC9	0.67	0.027	38.939	***			

Note: SIES = School-Based Intelligent Environment Support; AI-TS = AI Technological Support; AI-SE = AI Self-Efficacy; SRL = Self-Regulated Learning; PTIC = Pre-service Teachers’ Innovative Competence; *** *p* < 0.001.

**Table 3:** Correlation Coefficients and Discriminant Validity Test.

Variable	*M*	*SD*	SIES	AI-TS	AI-SE	SRL	PTIC
SIES	3.691	0.667	0.840				
AI-TS	3.979	0.625	0.329 **	0.827			
AI-SE	3.848	0.613	0.373 **	0.612 **	0.843		
SRL	3.834	0.606	0.447 **	0.481 **	0.536 **	0.820	
PTIC	3.793	0.596	0.541 **	0.539 **	0.615 **	0.621 **	0.792

Note: ** *p* < 0.01; the diagonal represents the square root of the average variance extracted (AVE) of the five variables; the lower triangular area represents the Pearson correlation coefficients among the five variables. SIES = School-Based Intelligent Environment Support; AI-TS = AI Technological Support; AI-SE = AI Self-Efficacy; SRL = Self-Regulated Learning; PTIC = Pre-service Teachers’ Innovative Competence.

**Table 4:** Quantile-Specific Regression Results.

Variable	Quantile								
	0.10	0.20	0.30	0.40	0.50	0.60	0.70	0.80	0.90
Constant	0.639 *** (0.121)	0.508 *** (0.085)	0.573 *** (0.060)	0.176 *** (0.037)	0.164 *** (0.030)	0.481 *** (0.035)	0.752 *** (0.040)	1.000 *** (0.111)	1.704 *** (0.124)
AI-TS	0.278 *** (0.027)	0.279 *** (0.019)	0.222 *** (0.013)	0.323 *** (0.008)	0.424 *** (0.007)	0.463 *** (0.008)	0.474 *** (0.010)	0.491 *** (0.029)	0.407 *** (0.036)
SIES	0.417 *** (0.026)	0.515 *** (0.018)	0.587 *** (0.011)	0.618 *** (0.007)	0.535 *** (0.006)	0.417 *** (0.008)	0.353 *** (0.010)	0.308 *** (0.027)	0.267 *** (0.033)
*R* ^2^	0.201	0.322	0.352	0.331	0.287	0.238	0.202	0.235	0.264

Note: SIES = School-Based Intelligent Environment Support; AI-TS = AI Technological Support; *** *p* < 0.001; values in parentheses represent standard errors (SE).

**Table 5:** Standardized Path Coefficients of the Model.

Model Path	Std Estimate	*SE*	*CR*	*p*
AI-TS→PTIC	0.128	0.019	6.147	***
AI-TS→AI-SE	0.609	0.020	31.973	***
AI-TS→SRL	0.423	0.019	22.347	***
SIES→PTIC	0.251	0.013	15.302	***
SIES→AI-SE	0.176	0.015	10.860	***
SIES→SRL	0.338	0.016	18.652	***
AI-SE→PTIC	0.329	0.017	16.512	***
SRL→PTIC	0.313	0.017	16.744	***

Note: SIES = School-Based Intelligent Environment Support; AI-TS = AI Technological Support; AI-SE = AI Self-Efficacy; SRL = Self-Regulated Learning; PTIC = Pre-service Teachers’ Innovative Competence; *** *p* < 0.001.

**Table 6:** Direct, Mediating, and Total Effects of the Model.

Independent Variable	Effect Type	Point Estimate	Coefficient Product		Bias-Corrected 95%CI		Percentile 95%CI		*p*
			*SE*	*Z*	Lower	Upper	Lower	Upper	
AI-TS	Indirect: AI-TS→AI-SE→PTIC	0.179	0.017	10.529	0.148	0.214	0.147	0.213	0.000
	Indirect: AI-TS→SRL→PTIC	0.119	0.014	8.500	0.092	0.147	0.092	0.147	0.000
	Diff1	0.061	0.022	2.773	0.017	0.104	0.017	0.104	0.007
	Direct: AI-TS→PTIC	0.115	0.024	4.792	0.069	0.162	0.069	0.162	0.000
	Total: AI-TS→AI-SE→PTIC	0.294	0.025	11.760	0.246	0.345	0.246	0.345	0.000
	Total: AI-TS→SRL→PTIC	0.233	0.025	9.320	0.185	0.284	0.185	0.284	0.000
SIES	Indirect: SIES→AI-SE→PTIC	0.045	0.008	5.625	0.032	0.062	0.031	0.062	0.000
	Indirect: SIES→SRL→PTIC	0.083	0.011	7.545	0.063	0.107	0.062	0.106	0.000
	Diff2	−0.038	0.013	−2.923	−0.066	−0.012	−0.065	−0.012	0.005
	Direct: SIES→PTIC	0.197	0.019	10.368	0.162	0.237	0.161	0.235	0.000
	Total: SIES→AI-SE→PTIC	0.242	0.021	11.524	0.204	0.285	0.203	0.284	0.000
	Total: SIES→SRL→PTIC	0.280	0.020	14.000	0.242	0.321	0.241	0.320	0.000

Note: SIES = School-Based Intelligent Environment Support; AI-TS = AI Technological Support; AI-SE = AI Self-Efficacy; SRL = Self-Regulated Learning; PTIC = Pre-service Teachers’ Innovative Competence.

**Table 7:** Summary of Hypothesis Testing Results.

Hypothesis	Hypothesis Statement	Result
H1	AI technological support is positively associated with pre-service teachers’ innovative competence.	Supported
H2	School-based intelligent environment support is positively associated with pre-service teachers’ innovative competence.	Supported
H3	AI self-efficacy mediates the relationship between AI technological support and pre-service teachers’ innovative competence.	Supported
H4	AI self-efficacy mediates the relationship between school-based intelligent environment support and pre-service teachers’ innovative competence.	Supported
H5	Self-regulated learning mediates the relationship between AI technological support and pre-service teachers’ innovative competence.	Supported
H6	Self-regulated learning mediates the relationship between school-based intelligent environment support and pre-service teachers’ innovative competence.	Supported
H7	The association of AI technological support with pre-service teachers’ innovative competence differs from that of school-based intelligent environment support.	Supported

## Data Availability

The data that support the findings of this study are available on request from the corresponding author.

## Appendix A

**Table A1:** Measurement Items and English Back-Translations.

Variable	Items	Chinese Item (Original)	English Translation (Back-Translated)
SIES	SIES1	我所在学校有足够资源支持AI技术应用于学生学习	My school has sufficient resources to support the application of AI technology in student learning.
	SIES2	学校资源能满足我在学习中使用AI技术的需求	School resources meet my needs for using AI technology in my studies.
	SIES3	我所在学校建设的智能教育平台功能齐全	The intelligent education platform built by my school has comprehensive functionalities.
	SIES4	我所在学校建设的智能教育平台具有丰富学习资源	The intelligent education platform built by my school has rich learning resources.
	SIES5	我所在学校建立了AI赋能学习的校内外合作网络	My school has established internal and external collaboration networks for AI-enabled learning.
	SIES6	我所在学校鼓励学生合作探索AI使用策略	My school encourages students to collaboratively explore AI usage strategies.
AI-TS	AITS1	我重视AI的有效利用	I value the effective utilization of AI.
	AITS2	我会优先考虑使用AI的创新学习与实践	I prioritize innovative learning and practice using AI.
	AITS3	使用AI将提升我的总体效率	Using AI will improve my overall efficiency.
	AITS4	使用AI能帮助我简化日常任务	Using AI helps me simplify daily tasks.
	AITS5	使用AI能帮助我从多学科中受益	Using AI helps me benefit from multiple disciplines.
AI-SE	AISE1	我坚信教师应积极学习AI技术以辅助教学	I firmly believe that teachers should actively learn AI technologies to assist teaching.
	AISE2	我相信AI技术可以促进教学设计多元化	I believe AI technology can promote diversified instructional design.
	AISE3	我希望利用AI技术进行教学创新	I hope to utilize AI technology for teaching innovation.
	AISE4	我认为利用AI技术辅助教学有利于提升教学效果	I think using AI technology to assist teaching is beneficial for improving instructional effectiveness.
	AISE5	我对AI技术辅助教学的应用效果持肯定态度	I hold a positive attitude towards the application effects of AI-assisted teaching.
	AISE6	我认为AI技术不会削弱教师的知识权威	I do not think AI technology will weaken teachers’ knowledge authority.
SRL	SRL1	我会根据学习目标制定和调整学习计划	I formulate and adjust my learning plans according to my learning goals.
	SRL2	我在学习过程中能够监控自己进度并调整学习内容	I can monitor my progress and adjust learning content during the learning process.
	SRL3	我能够识别并使用有效的学习策略来提高学习效率	I can identify and use effective learning strategies to improve learning efficiency.
	SRL4	我能够调整好自己情绪以保持积极的学习态度	I can regulate my emotions to maintain a positive learning attitude.
	SRL5	我能够主动寻求外部资源以支持我的学习情况	I can proactively seek external resources to support my learning.
PTIC	PTIC1	我愿意将AI创新地融入教育教学中	I am willing to innovatively integrate AI into education and teaching.
	PTIC2	我愿意运用AI开创教学新思路	I am willing to use AI to pioneer new teaching ideas.
	PTIC3	我对AI融入创新的教学过程充满信心	I am confident about integrating AI into innovative teaching processes.
	PTIC4	我准备运用AI培养学生数智素养	I am prepared to use AI to cultivate students’ digital intelligence literacy.
	PTIC5	我准备运用AI培养学生个性化学习能力	I am prepared to use AI to cultivate students’ personalized learning abilities.
	PTIC6	我准备运用AI优化教学内容结构	I am prepared to use AI to optimize the structure of teaching content.
	PTIC7	我经常使用AI支持的教学创新方法	I frequently use AI-supported teaching innovation methods.
	PTIC8	我经常使用AI支持的教学创新策略	I frequently use AI-supported teaching innovation strategies.
	PTIC9	我经常利用AI创新教学研究方式	I frequently utilize AI to innovate teaching research methods.

Note: SIES = School-Based Intelligent Environment Support; AI-TS = AI Technological Support; AI-SE = AI Self-Efficacy; SRL = Self-Regulated Learning; PTIC = Pre-service Teachers’ Innovative Competence; All items were measured using a 5-point Likert scale ranging from 1 (Strongly Disagree) to 5 (Strongly Agree). The survey was administered in Mandarin Chinese; English translations are provided for review.
